# The chalcone derivative (*E*)-1-(4-fluoro­phen­yl)-3-(4-hy­droxy-3-meth­oxy­phen­yl)prop-2-en-1-one monohydrate

**DOI:** 10.1107/S160053681300696X

**Published:** 2013-03-23

**Authors:** Florastina Payton-Stewart, Subramanya Ravi Kiran Pingali, James P. Donahue

**Affiliations:** a Department of Chemistry, Xavier University of New Orleans, 1 Drexel Drive, Box 114, New Orleans, Louisiana 70125, USA; b Department of Chemistry, Xavier University of New Orleans, 1 Drexel Drive, Box 22, New Orleans, Louisiana 70125, USA; c Department of Chemistry, Tulane University, 6400 Freret Street, New Orleans, Louisiana 70118-5698, USA

## Abstract

The title compound, C_16_H_13_FO_3_·H_2_O, has a *cis* disposition of the carbonyl and olefin bonds about the enone single bond. The arene rings are inclined to one another by 10.05 (6) Å. In the crystal, mol­ecules are linked *via* O—H⋯O hydrogen bonds involving the water mol­ecules, forming loops which are, in turn, linked *via* O—H.·O and C—H⋯F hydrogen bonds, forming sheets lying parallel to (103). These networks are linked *via* π–π inter­actions [centroid–centroid distance = 3.641 (1) Å] involving inversion-related 4-fluoro­phenyl and 4-hy­droxy-3-meth­oxy­phenyl rings.

## Related literature

For background information on the biological activity of chalcones, see: Anto *et al.* (1995 ▶); Calliste *et al.* (2001 ▶); Nowakowska (2007 ▶); Kontogiorgis *et al.* (2008 ▶); Ducki (2009 ▶); Batovska & Todorova (2010 ▶); Batovska & Parushev (2010 ▶); Gupta *et al.* (2010 ▶); Varinska *et al.* (2010 ▶); Katsori & Hadjipavlou-Litina (2011 ▶); Orlikova, *et al.* (2011 ▶); Yadav *et al.* (2011 ▶); Kathiravan *et al.* (2012 ▶); Sahu *et al.* (2012 ▶). For related chalcone structures, see: Rabinovich (1970 ▶); Ohkura *et al.* (1973 ▶); Hunter & Sanders (1990 ▶); Arai *et al.* (1994 ▶); Wu *et al.* (2006 ▶); Teh *et al.* (2006 ▶); Yathirajan *et al.* (2006 ▶, 2007 ▶); Butcher *et al.* (2007 ▶); Hayashi *et al.* (2009 ▶).
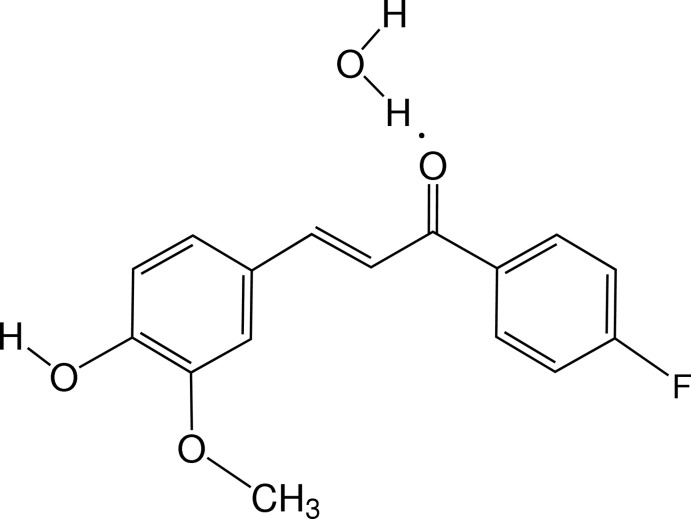


## Experimental

### 

#### Crystal data

C_16_H_13_FO_3_·H_2_O
*M* *_r_* = 290.29
Monoclinic, 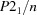

*a* = 9.787 (2) Å
*b* = 10.993 (3) Å
*c* = 12.781 (3) Å
β = 95.722 (4)°
*V* = 1368.2 (5) Å^3^
*Z* = 4
Mo *K*α radiation
μ = 0.11 mm^−1^
*T* = 100 K
0.34 × 0.27 × 0.21 mm

#### Data collection

Bruker SMART APEX CCD diffractometer
Absorption correction: multi-scan (*SADABS*; Sheldrick, 2009 ▶) *T*_min_ = 0.845, *T*_max_ = 0.978
11909 measured reflections
3203 independent reflections
2855 reflections with *I* > 2σ(*I*)
*R*_int_ = 0.022

#### Refinement

*R*[*F* ^2^ > 2σ(*F* ^2^)] = 0.041
*wR*(*F* ^2^) = 0.110
*S* = 1.02
3203 reflections
250 parameters
All H-atom parameters refined
Δρ_max_ = 0.37 e Å^−3^
Δρ_min_ = −0.22 e Å^−3^

### 

Data collection: *APEX2* (Bruker, 2010 ▶); cell refinement: *SAINT* (Bruker, 2010 ▶); data reduction: *SAINT*; program(s) used to solve structure: *SHELXS97* (Sheldrick, 2008 ▶); program(s) used to refine structure: *SHELXL97* (Sheldrick, 2008 ▶); molecular graphics: *XP* in *SHELXTL* (Sheldrick, 2008 ▶); software used to prepare material for publication: *SHELXTL*.

## Supplementary Material

Crystal structure: contains datablock(s) I, global. DOI: 10.1107/S160053681300696X/pk2468sup1.cif

Structure factors: contains datablock(s) I. DOI: 10.1107/S160053681300696X/pk2468Isup2.hkl

Supplementary material file. DOI: 10.1107/S160053681300696X/pk2468Isup3.cml

Additional supplementary materials:  crystallographic information; 3D view; checkCIF report

## Figures and Tables

**Table 1:** Hydrogen-bond geometry (Å, °)

*D*—H⋯*A*	*D*—H	H⋯*A*	*D*⋯*A*	*D*—H⋯*A*
O2—H2⋯O4^i^	0.91 (2)	1.74 (2)	2.6479 (14)	173.5 (18)
O4—H4*A*⋯O1	0.88 (2)	1.90 (2)	2.7672 (15)	173 (2)
O4—H4*B*⋯O2^ii^	0.83 (3)	2.17 (3)	2.8485 (15)	139 (2)
O4—H4*B*⋯O3^ii^	0.83 (3)	2.38 (3)	3.1283 (15)	151 (2)
C8—H8⋯F1^iii^	0.94 (2)	2.48 (2)	3.3931 (17)	164 (1)

Symmetry codes: (i) 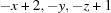; (ii) 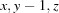; (iii) 
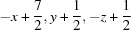
.

**Fig. 1.**
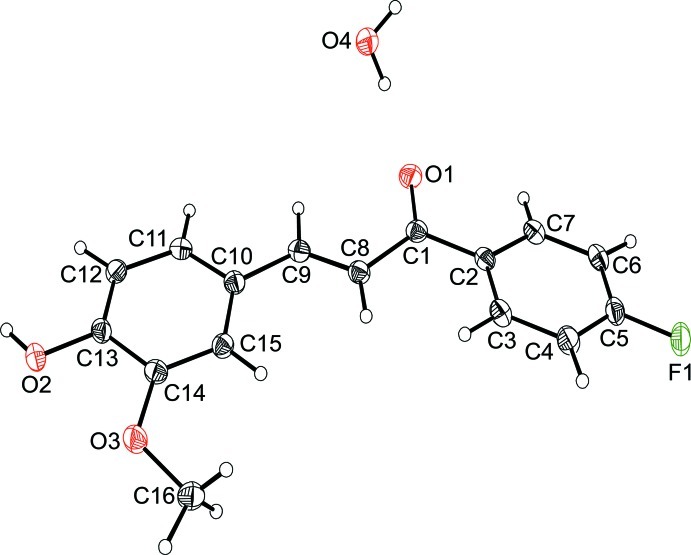
Thermal ellipsoid plot drawn at the 50% probability level for (E)-1-(4-fluorophenyl)-3-(4-hydroxy-3-methoxyphenyl)prop-2-ene-1-one monohydrate.

**Fig. 2.**
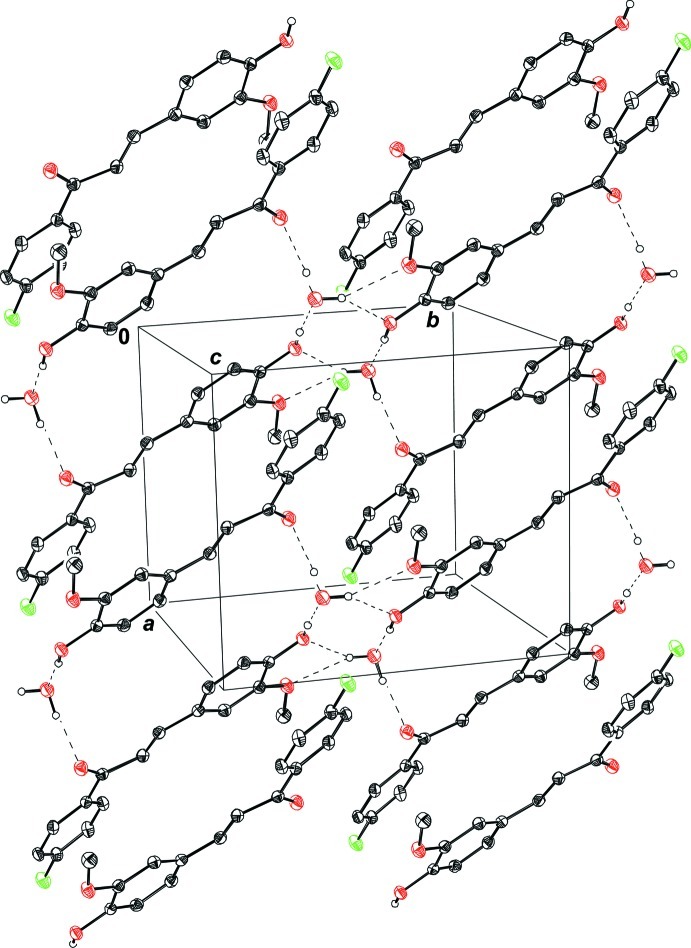
Partial packing diagram showing the two-dimensional sheet network of molecules in the ab plane, the formation of which is governed by the occurrence of numerous hydrogen bonds with co-crystallized water molecules. Oxygen atoms are illustrated in red and fluorine atoms in light green. Hydrogen bonds are depicted with dashed lines.

## supplementary crystallographic information

### Comment

The chalcones, or 1,3-diaryl-2-propene-1-ones, constitute a relatively simple but pharmacologically important class of organic compounds with reported biological activity as antifungal agents (Kathiravan *et al.*, 2012), antimicrobial (e.g., bacteria and protozoa) agents (Nowakowska, 2007; Gupta *et al.*, 2010; Sahu *et al.*, 2012), anti-inflammatory agents (Nowakowska, 2007; Sahu *et al.*, 2012; Katsori & Hadjipavlou-Litina, 2011; Batovska & Todorova, 2010; Kontogiorgis *et al.*, 2008) and potential cancer therapeutics (Yadav *et al.*, 2011; Orlikova, *et al.*, 2011; Batovska & Parushev, 2010; Varinska *et al.*, 2010; Ducki, 2009). Variants bearing methoxy and hydroxy ring substituents have in some instances been observed to display enhanced efficacy (Calliste *et al.*, 2001; Anto *et al.*, 1995), possibly because of improved water solubility, improved binding ability to *in vivo* substrate(s) via hydrogen bond formation, or both. The ease with which a diverse array of chalcone derivatives can be synthesized and their usefulness for the further synthesis of other, biologically important heterocyclic compounds continue to motivate research involving their preparation and the evaluation of their properties. In the course of our own studies of chalcone derivatives, we have prepared (*E*)-1-(4-fluorophenyl)-3-(4-hydroxy-3-methoxyphenyl)prop-2-ene-1-one (I, Scheme 1) in a form suitable for a structural characterization by X-ray diffraction, the results of which are herein reported.

The title compound shows near planarity in the crystalline state, the twist angle between the aromatic rings being 10.05 (6)°. The greatest departure from the mean plane defined by all the nonhydrogen atoms is by C3, which deviates by 0.336 (1) Å from the plane of the molecule in Fig. 1. A *cis* disposition of the olefinic and carbonyl functional groups about the C1-C8 single bond is observed, which is the conformation found for chalcone itself in the crystalline state (Rabinovich, 1970; Ohkura *et al.*, 1973; Arai *et al.*, 1994; Wu *et al.*, 2006) and for most of its simple derivatives. The *trans* orientation of olefinic and carbonyl functional groups about the enone single bond is observed less frequently (Teh *et al.*, 2006; Yathirajan, *et al.*, 2006; Yathirajan, *et al.*, 2007; Butcher *et al.*, 2007) in the crystalline state for chalcones, possibly because it is less conducive to stabilizing intermolecular π-π stacking interactions (see below). All other intramolecular structural parameters observed for I are typical of the compound type.

The packing arrangement of I can be described first by the association of two molecules at their 4-hydroxy-3-methoxy phenyl ends around an inversion center that occurs on the *bc* faces of the cell (Fig. 2). This arrangement is mediated by the presence of two water molecules, one canted slightly above the inversion center and the other below, each providing for four hydrogen bonds. One hydrogen atom from the water molecules is disposed halfway between the 3-methoxy and 4-hydroxy oxygen atoms of one molecule such that it serves as hydrogen bond donor to both (Fig. 2). The 4-hydroxy group of the molecule on the opposite side of the inversion center in turn serves as hydrogen bond donor to this same H_2_O molecule. This pattern of hydrogen bonds is replicated by the inversion center between the two molecules of I, thereby providing a network of six hydrogen bonds at this "head-to-head" interface of two molecules. The second hydrogen atom of H_2_O is directed away from the network just described and forms a hydrogen bond with the propen-2-one oxygen atom of a neighboring molecule (O1). The effect of this last hydrogen bond is to produce a two dimensional sheet arrangement of molecules that parallels the *ab* plane. This packing arrangement is reinforced by apparent π-π stacking interactions between the 4-F-phenyl group of one molecule and the 4-hydroxy-3-methoxy phenyl group of another (Fig. 2). The distance between the centers of these aromatic rings is 3.641 (1) Å, and at the point of closest approach (C7···C13) the two rings are 3.326 (2) Å apart. These values are within the range of distances (3.4-3.6 Å (Hunter & Sanders, 1990), 3.4-3.8 Å (Hayashi *et al.*, 2009) that has been reported as indicative of π-π stacking. A second sheet network of molecules of I (not shown), is created by applying the glide plane operation to the sheets illustrated.

### Experimental

(*E*)-1-(4-fluorophenyl)-3-(4-hydroxy-3-methoxyphenyl)prop-2-ene-1-one (I). Solid potassium hydroxide (20 g, 0.36 mol) was added to a mixture of vanillin (3.0 g, 0.020 mol) and 4-fluoroacetophenone (2.8 g, 0.020 mol) in 40 mL of methanol and 20 mL of H_2_O. The resulting solution was refluxed for 15 minutes and cooled in an ice bath. The reaction mixture was then diluted with 200 mL of H_2_O and stored in the refrigerator overnight. The precipitated yellow solid was collected by vacuum filtration and dried. The crude material was then purified via flash chromatography on silica gel with 20:80 ethyl acetate:hexanes as the eluting solvent to yield a dark yellow solid (I) in 35% yield. ^1^H NMR (CDCl_3_) δ (in ppm): 3.87 (s, 3H, -OC*H*_3_), 5.95 (s, 1H, -O*H*), 6.95 (d, 1H, Ar-5'*H*), 7.15 (d, 2H, Ar-3", 5"*H*), 7.17 (d, 1H, Ar-6'*H*), 7.25 (d, 1H, =C*H*_a_), 7.31 (s, 1H, Ar-2'*H*), 7.75 (d, 1H, =C*H*_b_), 8.04 (m, 2H, Ar-2",6"*H*). ^13^C NMR (acetone) δ (in ppm): 56.23 (O*C*H_3_), 110.27 (Ar-*C*2'), 115.15 (Ar-*C*3", *C*5"), 115.78 (Ar-*C*5'), 116.00 (Ar-*C*6'), 119.39 (Ar-*C*1'), 123.68 (=*C*_b_), 127.53 (Ar-*C*2",*C*6"), 131.18 (Ar-*C*1"), 145.72 (Ar-*C*4'), 147.06 (=*C*_a_), 148.65 (Ar-*C*3'), 166.96 (Ar-*C*4"), 189.21 (*C*=O). Diffraction quality, pale yellow, needle-shaped crystals were obtained by slow cooling of a warm solution in 1:1 H_2_O:EtOH.

### Refinement

Hydrogen atoms were identified in the later difference maps, and their positions were refined with isotropic displacement parameters that were approximately 1.2–1.5 times (for carbon) or 2.0 times (for oxygen) those of the atoms to which they were attached.

### Crystal data

**Table:**

C_16_H_13_FO_3_·H_2_O	*F*(000) = 608
*M**_r_* = 290.29	*D*_x_ = 1.409 Mg m^−^^3^
Monoclinic, *P*2_1_/*n*	Mo *K*α radiation, λ = 0.71073 Å
Hall symbol: -P 2yn	Cell parameters from 7532 reflections
*a* = 9.787 (2) Å	θ = 28.3–2.5°
*b* = 10.993 (3) Å	µ = 0.11 mm^−^^1^
*c* = 12.781 (3) Å	*T* = 100 K
β = 95.722 (4)°	Needle, pale yellow
*V* = 1368.2 (5) Å^3^	0.34 × 0.27 × 0.21 mm
*Z* = 4	

### Data collection

**Table:**

Bruker SMART APEX CCD diffractometer	3203 independent reflections
Radiation source: fine-focus sealed tube	2855 reflections with *I* > 2σ(*I*)
Graphite monochromator	*R*_int_ = 0.022
φ and ω scans	θ_max_ = 28.3°, θ_min_ = 2.5°
Absorption correction: multi-scan (*SADABS*; Sheldrick, 2009)	*h* = −13→12
*T*_min_ = 0.845, *T*_max_ = 0.978	*k* = −13→14
11909 measured reflections	*l* = −16→16

### Refinement

**Table:**

Refinement on *F*^2^	Primary atom site location: structure-invariant direct methods
Least-squares matrix: full	Secondary atom site location: difference Fourier map
*R*[*F*^2^ > 2σ(*F*^2^)] = 0.041	Hydrogen site location: inferred from neighbouring sites
*wR*(*F*^2^) = 0.110	All H-atom parameters refined
*S* = 1.02	*w* = 1/[σ^2^(*F*_o_^2^) + (0.0607*P*)^2^ + 0.5324*P*] where *P* = (*F*_o_^2^ + 2*F*_c_^2^)/3
3203 reflections	(Δ/σ)_max_ = 0.001
250 parameters	Δρ_max_ = 0.37 e Å^−^^3^
0 restraints	Δρ_min_ = −0.22 e Å^−^^3^

### Special details

**Table:**

Experimental. The diffraction data were obtained from 3 sets of 606 frames, each of width 0.3 ° in ω, collected at φ = 0.00, 120.00 and 240.00 °. The scan time was 30 sec/frame.
Geometry. All e.s.d.'s (except the e.s.d. in the dihedral angle between two l.s. planes) are estimated using the full covariance matrix. The cell e.s.d.'s are taken into account individually in the estimation of e.s.d.'s in distances, angles and torsion angles; correlations between e.s.d.'s in cell parameters are only used when they are defined by crystal symmetry. An approximate (isotropic) treatment of cell e.s.d.'s is used for estimating e.s.d.'s involving l.s. planes.
Refinement. Refinement of *F*^2^ against ALL reflections. The weighted *R*-factor *wR* and goodness of fit *S* are based on *F*^2^, conventional *R*-factors *R* are based on *F*, with *F* set to zero for negative *F*^2^. The threshold expression of *F*^2^ > 2σ(*F*^2^) is used only for calculating *R*-factors(gt) *etc*. and is not relevant to the choice of reflections for refinement. *R*-factors based on *F*^2^ are statistically about twice as large as those based on *F*, and *R*-factors based on ALL data will be even larger.

### Fractional atomic coordinates and isotropic or equivalent isotropic displacement parameters (Å^2^)

**Table:**

	*x*	*y*	*z*	*U*_iso_*/*U*_eq_	
F1	1.90355 (8)	−0.41277 (7)	0.21518 (6)	0.0284 (2)	
O1	1.40402 (9)	−0.31928 (8)	0.47673 (7)	0.0243 (2)	
O2	1.03808 (9)	0.36973 (8)	0.45389 (7)	0.0216 (2)	
H2	0.977 (2)	0.3730 (17)	0.5032 (16)	0.043 (5)*	
O3	1.23977 (10)	0.34671 (8)	0.33849 (7)	0.0237 (2)	
O4	1.14188 (10)	−0.39678 (10)	0.40629 (8)	0.0266 (2)	
H4A	1.227 (2)	−0.378 (2)	0.4266 (16)	0.052 (6)*	
H4B	1.141 (2)	−0.472 (2)	0.3984 (17)	0.063 (7)*	
C1	1.45724 (12)	−0.24717 (11)	0.41849 (9)	0.0184 (3)	
C2	1.57311 (12)	−0.28995 (11)	0.36004 (9)	0.0178 (2)	
C3	1.61894 (13)	−0.22551 (11)	0.27605 (10)	0.0210 (3)	
H3	1.5730 (17)	−0.1494 (16)	0.2507 (13)	0.031 (4)*	
C4	1.72921 (14)	−0.26744 (12)	0.22595 (10)	0.0233 (3)	
H4	1.7621 (17)	−0.2243 (16)	0.1683 (13)	0.030 (4)*	
C5	1.79286 (13)	−0.37355 (12)	0.26222 (10)	0.0215 (3)	
C6	1.75010 (13)	−0.44096 (11)	0.34390 (10)	0.0211 (3)	
H6	1.7980 (16)	−0.5140 (16)	0.3672 (12)	0.029 (4)*	
C7	1.63853 (13)	−0.39926 (11)	0.39241 (10)	0.0201 (3)	
H7	1.6074 (16)	−0.4430 (14)	0.4491 (13)	0.024 (4)*	
C8	1.41072 (13)	−0.12016 (11)	0.40715 (10)	0.0195 (3)	
H8	1.4531 (16)	−0.0693 (15)	0.3609 (13)	0.027 (4)*	
C9	1.31350 (12)	−0.07855 (11)	0.46511 (10)	0.0189 (3)	
H9	1.2769 (16)	−0.1366 (15)	0.5109 (12)	0.024 (4)*	
C10	1.25059 (12)	0.04162 (11)	0.46436 (9)	0.0182 (2)	
C11	1.14937 (13)	0.06159 (11)	0.53213 (10)	0.0196 (3)	
H11	1.1290 (16)	−0.0018 (16)	0.5787 (13)	0.028 (4)*	
C12	1.07706 (12)	0.17051 (11)	0.53043 (10)	0.0199 (3)	
H12	1.0059 (16)	0.1842 (14)	0.5779 (12)	0.024 (4)*	
C13	1.10616 (12)	0.26192 (11)	0.46193 (9)	0.0182 (3)	
C14	1.21398 (12)	0.24626 (11)	0.39687 (9)	0.0187 (3)	
C15	1.28376 (12)	0.13679 (11)	0.39695 (10)	0.0189 (3)	
H15	1.3551 (16)	0.1271 (15)	0.3518 (13)	0.026 (4)*	
C16	1.36498 (14)	0.34589 (13)	0.28868 (11)	0.0250 (3)	
H16A	1.4443 (17)	0.3331 (15)	0.3422 (13)	0.028 (4)*	
H16C	1.3620 (16)	0.2806 (14)	0.2335 (12)	0.023 (4)*	
H16B	1.3695 (17)	0.4287 (15)	0.2550 (13)	0.030 (4)*	

### Atomic displacement parameters (Å^2^)

**Table:**

	*U*^11^	*U*^22^	*U*^33^	*U*^12^	*U*^13^	*U*^23^
F1	0.0297 (4)	0.0264 (4)	0.0307 (4)	0.0078 (3)	0.0101 (3)	−0.0034 (3)
O1	0.0235 (5)	0.0194 (4)	0.0306 (5)	−0.0001 (4)	0.0062 (4)	0.0052 (4)
O2	0.0219 (4)	0.0178 (4)	0.0252 (5)	0.0039 (3)	0.0035 (4)	−0.0002 (3)
O3	0.0272 (5)	0.0195 (5)	0.0254 (5)	0.0023 (4)	0.0075 (4)	0.0047 (3)
O4	0.0229 (5)	0.0207 (5)	0.0369 (6)	0.0017 (4)	0.0063 (4)	0.0015 (4)
C1	0.0169 (5)	0.0181 (6)	0.0195 (6)	−0.0012 (4)	−0.0010 (4)	0.0003 (4)
C2	0.0186 (6)	0.0152 (5)	0.0191 (6)	−0.0001 (4)	−0.0006 (4)	−0.0014 (4)
C3	0.0260 (6)	0.0160 (6)	0.0209 (6)	0.0026 (5)	0.0017 (5)	−0.0001 (4)
C4	0.0307 (7)	0.0188 (6)	0.0213 (6)	0.0017 (5)	0.0070 (5)	0.0005 (5)
C5	0.0228 (6)	0.0202 (6)	0.0218 (6)	0.0028 (5)	0.0031 (5)	−0.0064 (5)
C6	0.0245 (6)	0.0155 (6)	0.0224 (6)	0.0043 (5)	−0.0018 (5)	−0.0021 (4)
C7	0.0243 (6)	0.0158 (6)	0.0199 (6)	−0.0001 (5)	0.0014 (5)	−0.0001 (5)
C8	0.0194 (6)	0.0170 (6)	0.0219 (6)	−0.0007 (5)	0.0013 (5)	0.0014 (5)
C9	0.0189 (6)	0.0175 (6)	0.0198 (6)	−0.0016 (4)	−0.0004 (5)	0.0011 (4)
C10	0.0178 (5)	0.0180 (6)	0.0183 (6)	−0.0003 (4)	−0.0005 (4)	−0.0016 (4)
C11	0.0201 (6)	0.0183 (6)	0.0206 (6)	−0.0025 (5)	0.0021 (5)	0.0007 (5)
C12	0.0176 (6)	0.0206 (6)	0.0218 (6)	−0.0008 (4)	0.0031 (5)	−0.0022 (5)
C13	0.0172 (5)	0.0167 (6)	0.0201 (6)	0.0009 (4)	−0.0011 (4)	−0.0031 (4)
C14	0.0199 (6)	0.0179 (6)	0.0179 (6)	−0.0013 (4)	0.0003 (5)	0.0004 (4)
C15	0.0185 (6)	0.0201 (6)	0.0183 (6)	0.0000 (4)	0.0024 (4)	−0.0008 (4)
C16	0.0262 (7)	0.0247 (7)	0.0247 (6)	−0.0011 (5)	0.0064 (5)	0.0046 (5)

### Geometric parameters (Å, º)

**Table:**

F1—C5	1.3604 (14)	C6—H6	0.962 (17)
O1—C1	1.2374 (15)	C7—H7	0.945 (16)
O2—C13	1.3585 (15)	C8—C9	1.3430 (18)
O2—H2	0.91 (2)	C8—H8	0.939 (17)
O3—C14	1.3702 (15)	C9—C10	1.4572 (17)
O3—C16	1.4361 (16)	C9—H9	0.960 (16)
O4—H4A	0.88 (2)	C10—C11	1.3968 (17)
O4—H4B	0.83 (3)	C10—C15	1.4135 (17)
C1—C8	1.4712 (17)	C11—C12	1.3899 (18)
C1—C2	1.4947 (17)	C11—H11	0.950 (17)
C2—C3	1.3969 (18)	C12—C13	1.3811 (18)
C2—C7	1.4043 (17)	C12—H12	0.980 (16)
C3—C4	1.3874 (18)	C13—C14	1.4175 (17)
C3—H3	0.989 (17)	C14—C15	1.3837 (17)
C4—C5	1.3807 (18)	C15—H15	0.955 (16)
C4—H4	0.959 (17)	C16—H16A	0.992 (17)
C5—C6	1.3788 (19)	C16—H16C	1.005 (16)
C6—C7	1.3863 (18)	C16—H16B	1.009 (17)
			
C13—O2—H2	109.5 (12)	C8—C9—C10	128.85 (12)
C14—O3—C16	116.62 (10)	C8—C9—H9	116.0 (9)
H4A—O4—H4B	106 (2)	C10—C9—H9	115.1 (9)
O1—C1—C8	121.50 (11)	C11—C10—C15	118.81 (11)
O1—C1—C2	119.00 (11)	C11—C10—C9	117.62 (11)
C8—C1—C2	119.48 (11)	C15—C10—C9	123.54 (11)
C3—C2—C7	119.24 (11)	C12—C11—C10	121.14 (12)
C3—C2—C1	122.83 (11)	C12—C11—H11	120.2 (10)
C7—C2—C1	117.93 (11)	C10—C11—H11	118.7 (10)
C4—C3—C2	120.69 (12)	C13—C12—C11	120.00 (11)
C4—C3—H3	118.6 (10)	C13—C12—H12	119.0 (9)
C2—C3—H3	120.7 (10)	C11—C12—H12	121.0 (9)
C5—C4—C3	118.12 (12)	O2—C13—C12	123.52 (11)
C5—C4—H4	120.1 (10)	O2—C13—C14	116.73 (11)
C3—C4—H4	121.8 (10)	C12—C13—C14	119.74 (11)
F1—C5—C6	118.65 (11)	O3—C14—C15	125.71 (11)
F1—C5—C4	118.15 (12)	O3—C14—C13	114.20 (10)
C6—C5—C4	123.19 (12)	C15—C14—C13	120.09 (11)
C5—C6—C7	118.23 (12)	C14—C15—C10	120.06 (11)
C5—C6—H6	120.5 (9)	C14—C15—H15	119.1 (10)
C7—C6—H6	121.2 (9)	C10—C15—H15	120.8 (10)
C6—C7—C2	120.49 (12)	O3—C16—H16A	109.8 (9)
C6—C7—H7	120.2 (10)	O3—C16—H16C	110.4 (9)
C2—C7—H7	119.3 (9)	H16A—C16—H16C	110.4 (13)
C9—C8—C1	119.88 (11)	O3—C16—H16B	105.0 (10)
C9—C8—H8	121.7 (10)	H16A—C16—H16B	110.9 (14)
C1—C8—H8	118.4 (10)	H16C—C16—H16B	110.1 (13)

### Hydrogen-bond geometry (Å, º)

**Table:**

*D*—H···*A*	*D*—H	H···*A*	*D*···*A*	*D*—H···*A*
O2—H2···O4^i^	0.91 (2)	1.74 (2)	2.6479 (14)	173.5 (18)
O4—H4*A*···O1	0.88 (2)	1.90 (2)	2.7672 (15)	173 (2)
O4—H4*B*···O2^ii^	0.83 (3)	2.17 (3)	2.8485 (15)	139 (2)
O4—H4*B*···O3^ii^	0.83 (3)	2.38 (3)	3.1283 (15)	151 (2)
C8—H8···F1^iii^	0.94 (2)	2.48 (2)	3.3931 (17)	164 (1)

Symmetry codes: (i) −*x*+2, −*y*, −*z*+1; (ii) *x*, *y*−1, *z*; (iii) −*x*+7/2, *y*+1/2, −*z*+1/2.
